# Long noncoding RNA TINCR is a novel regulator of human bronchial epithelial cell differentiation state

**DOI:** 10.14814/phy2.14727

**Published:** 2021-02-01

**Authors:** Norihito Omote, Koji Sakamoto, Qin Li, Jonas C. Schupp, Taylor Adams, Farida Ahangari, Maurizio Chioccioli, Giuseppe DeIuliis, Naozumi Hashimoto, Yoshinori Hasegawa, Naftali Kaminski

**Affiliations:** ^1^ Pulmonary, Critical Care and Sleep Medicine Section Department of Internal Medicine Yale University School of Medicine New Haven CT USA; ^2^ Department of Respiratory Medicine Nagoya University Graduate School of Medicine Nagoya Japan; ^3^ Department of Respiratory Medicine National Hospital Organization Nagoya Medical Center Nagoya Japan

**Keywords:** bronchial epithelial cell differentiation, lncRNA, Staufen1, TINCR

## Abstract

Long‐noncoding RNAs (lncRNAs) have numerous biological functions controlling cell differentiation and tissue development. The knowledge about the role of lncRNAs in human lungs remains limited. Here we found the regulatory role of the terminal differentiation‐induced lncRNA (TINCR) in bronchial cell differentiation. RNA in situ hybridization revealed that TINCR was mainly expressed in bronchial epithelial cells in normal human lung. We performed RNA sequencing analysis of normal human bronchial epithelial cells (NHBECs) with or without TINCR inhibition and found the differential expression of 603 genes, which were enriched for cell adhesion and migration, wound healing, extracellular matrix organization, tissue development and differentiation. To investigate the role of TINCR in the differentiation of NHBECs, we employed air–liquid interface culture and 3D organoid formation assay. TINCR was upregulated during differentiation, loss of TINCR significantly induced an early basal‐like cell phenotype (TP63) and a ciliated cell differentiation (FOXJ1) in late phase and TINCR overexpression suppressed basal cell phenotype and the differentiation toward to ciliated cells. Critical regulators of differentiation such as SOX2 and NOTCH genes (NOTCH1, HES1, and JAG1) were significantly upregulated by TINCR inhibition and downregulated by TINCR overexpression. RNA immunoprecipitation assay revealed that TINCR was required for the direct bindings of Staufen1 protein to SOX2, HES1, and JAG1 mRNA. Loss of Staufen1 induced TP63, SOX2, NOTCH1, HES1, and JAG1 mRNA expressions, which TINCR overexpression suppressed partially. In conclusion, TINCR is a novel regular of bronchial cell differentiation, affecting downstream regulators such as SOX2 and NOTCH genes, potentially in coordination with Staufen1.

## Graphical abstract

Proposed model representing that TINCR together with STAU1 controls the expression of critical regulators of bronchial cell differentiation and maintains the normal differentiation state.

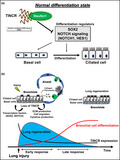

## 1 INTRODUCTION

Long noncoding RNAs (lncRNAs) are nonprotein coding RNA transcripts usually longer than 200 bases that play important roles in various biological processes by regulating gene expression at epigenetic, transcriptional, and posttranscriptional levels (Herriges et al., 2014; Jandura & Krause, 2017). In recent years, there has been increased interest in the role of lncRNAs in the lung, with evidence that they are involved in cell cycle, apoptosis, tissue development, and cellular differentiation (Grote et al., 2013; Herriges et al., 2014). Aberrant expressions of lncRNA has been observed and implicated in a variety of lung disease (Groot & Jin, 2018; Guo et al., 2019). Despite these advances, the molecular functions of many lung‐specific lncRNAs remain elusive, and more detailed functional studies are needed to clarify their biological roles.

The terminal differentiation‐induced lncRNA (TINCR) is a 3.7 kb lncRNA mainly expressed in the epidermis, placenta, esophagus, and the lung (Fagerberg et al., 2014). Previous studies reported that TINCR controls human epidermal differentiation and its altered expression has been observed in several cancers including lung cancer (Dong et al., 2018; Kretz et al., 2013; Zhang et al., 2016). TINCR has been shown to bind to Staufen1 (STAU1) protein and mediate stabilization of cell cycle and differentiation‐related mRNAs in epidermal cells (Kretz, 2013; Kretz et al., 2013; Xu et al., 2017). However, the cellular distribution and mechanistic role of TINCR in the lung are not well understood. Based on its altered expression in different epithelial cancers and differentiation of epithelial tissues, we hypothesized that TINCR may have a critical role in the function of the lung epithelium. Indeed, we found that TINCR expression was mainly localized to bronchial epithelial cells in the human lung, and loss of TINCR led to aberrant bronchial cell differentiation, with induction of ciliated cell phenotype in in vitro assay model. We also determined that TINCR regulates SOX2, and NOTCH signaling (HES1 and JAG1) mRNA at least in part together with STAU1 protein. Taken together, our data suggest that TINCR plays important role in maintaining the normal differentiation state of bronchial epithelial cells.

## 2 MATERIALS AND METHODS

### 2.1 Cells

Primary normal human bronchial epithelial cells (NHBECs) were purchased from Life Line Cell Technology (Fredefick, MD). NHBECs were maintained in BronchiaLife™ Basal Medium supplemented with 500 µg/ml HAS, 0.6 µM Linoleic Acid, 0.6 µg/ml Lecithin, 6 mM l‐glutamine,0.4% Extract P, 1 µM Epinephrine, 5 µg/ml Transferrin PS, 10 nM T3, 0.1 µg/ml Hydrocortisone, 5 ng/mL rhEGF, 5 µg·ml rhInsulin, 30 mg/ml Gentmycin, and 15 µg/ml Amphotericin B. In air–liquid interface (ALI) culture model, cells were seeded at passage 2–3 at a density of 1 × 10^5^ cells/cm^2^ on transwell inserts (Transwell, 12 wells, 0.4 µm, Cat. No 3460, Corning) in BronchiaLife™ Basal Medium. Cells were lifted to the ALI when confluent, apical media was aspirated, and basolateral media was substituted with BronchiaLife™ Air‐Liquid Interface Epithelial Differentiation Medium and changed every other day. In 3D organoid formation assay, cells were seeded at passage 2–3 at a density of 2.5 × 10^3^ cells/well on 96 well culture plate in BronchiaLife™ Air‐Liquid Interface Epithelial Differentiation Medium with 2% Growth Factor Reduced Matrigel (Corning). Each well was added 20 µl of 100% Growth Factor Reduced Matrigel (Corning) on the bottom and incubated for 15 min before cells were seeded. Medium was changed every 5 days.

### 2.2 Materials

Purified rabbit anti‐TP63 antibody was from Gene Tex (cat. No. GTX102425). Rabbit anti‐SOX2 antibody (cat. No 3579T), rabbit anti‐HES1 antibody (cat. No 11988S), rabbit anti‐JAG1 antibody (cat. No 2620T), rabbit anti‐NOTCH1 antibody (cat. No 3608S), and rabbit anti‐NOTCH2 antibody (cat. No 5732T) were from Cell Signaling Technology. Rabbit anti‐STAU1 antibody is from Proteintech (cat. No. 14225–1‐AP). Anti‐rabbit IgG HRP‐linked antibody (cat. No. GENA934) and Anti‐mouse IgG HRP‐linked antibody (cat. No. NXA931) were from Sigma Aldrich. Gout anti‐rabbit IgG (H + L) cross‐absorbed secondary antibody, FITC (cat. No. F‐2765) and Gout anti‐mouse IgG (H + L) cross‐absorbed secondary antibody, Texas Red‐X (cat. No. T‐6390) were from Thermo Fisher Scientific.

### 2.3 Lung tissues

Lung tissue samples were surgical remnants of biopsies or lung explants obtained through the National Disease Research Interchange and Yale University Pathology Tissue service. We analyzed RNA expression of the lungs from patients with controls (CTRL; *n* = 22), chronic obstructive pulmonary disease (COPD) (*n* = 44), and IPF (*n* = 23). These lungs are from the Lung Genomics Research Consortium (LGRC) https://www.atsjournals.org/doi/full/10.1164/rccm.201510‐2026OC.

### 2.4 Cell transfection

TINCR sequences were cloned into pcDNA.3.1‐EGFP vector. We conducted the transfections by using a Lipofectamine 2000 kit (Thermo Fisher Scientific). NHBECs were transfected with TINCR‐pcDNA3.1(+)‐EGFP or Blank‐pcDNA3.1(+)‐EGFP. At 96 h post‐transfection, cells were analyzed by qRT‐PCR and Western blot analysis. Transfection of small interfering RNA (siRNA) into NHBECs was carried out using Lipofectamine RNAiMAX according to the manufacturer's instructions. Two siRNAs targeting TINCR (siTINCR1 and siTINCR2) and a scramble siRNA (siSCR) use as negative control were synthesized. At 96 h post‐transfection, cells were analyzed by qRT‐PCR and Western blot analysis.

### 2.5 Quantitative real‐time PCR

TaqMan (Life Technologies, Thermo Scientific Inc.) was used according to the manufacturer's instructions, using b‐glucuronidase (GUSB) as an internal control and manufacturer's specific primers and probes. Relative gene expression was normalized to a value of 1.0 for the unstimulated control group. Fold change was calculated by taking the mean of the controls as baseline.

### 2.6 Western blot analysis

Western blot analysis of targeted proteins was performed as previously described (Tzouvelekis et al., 2017). Rabbit anti‐TP63 antibody, rabbit anti‐STAU1 antibody, rabbit anti‐SOX2 antibody, rabbit anti‐HES1 antibody, rabbit anti‐JAG1 antibody, rabbit anti‐NOTCH1 antibody, and rabbit anti‐NOTCH2 antibody were used as the primary antibodies, and immunostained bands were visualized with an appropriate HRP‐labeled anti‐primary antibody, followed by exposure to Clarity™ Western ECL Substrate (Bio‐Rad Laboratories).

### 2.7 Cell migration and proliferation assay

For cell migration assays, cells were cultured in 6‐well plates and grown to 100% confluence following transfection with SCR siRNA or TINCR siRNA. After being incubated at 37°C with 5% CO_2_ for 24 h, the confluent monolayer was scratched using a standard p200 pipette tip. The ability of cells to migrate into the wound area was estimated by comparing the distance traveled by the cells at the acellular front at 0 h and 24 h using light microscopy. For proliferation analysis, transfected cells were seeded on 96‐well plates at a density of 10,000 cells/well. Cells were analyzed 0, 24, and 72 h later by WST‐1 Assay Kit (cat. No ab65475, Abcam).

### 2.8 Immunofluorescence staining and confocal laser scanning microscopy

Cultured NHBEs on transwells and matrigel were briefly washed three times with PBS and fixed in 4% paraformaldehyde for 10 min. The cells were then permeabilized with 0.2% Triton X‐100 in PBS for 20 min and blocked in goat serum for 1 h at room temperature (RT). The cells were incubated with TP63 (Cat. No. V3815SAF, NSJ Bioreagents) and FOXJ1 (cat. No. 14‐9965‐80. Thermo Scientific, MA, USA) antibodies overnight at 4°C and subsequently incubated with FITC‐conjugated goat anti‐rabbit and Texas red conjugated goat anti‐mouse antibodies for 1 h. The cells were washed with PBS, and the nuclei were stained with Hoechst 33342 (Roche Molecular Biochemicals) for 5 min at RT. Immunofluorescence signals were analyzed determined by confocal laser scanning microscopy (Leica SP5 Confocal Microscope, Leica Microsystems Inc.).

### 2.9 RNA‐sequencing

We performed RNA transcriptome sequencing from control (siSCR) or TINCR‐depleted NHBECs using two independent siRNAs (siTINCR1 and siTINCR2). Total RNA was isolated from NHBECs transfected siSCR, siTINCR1, and siTINCR2. For library construction, 75 ng of RNA was amplified using the Ion Ampliseq™ kit for Chef DL8 (A29024) according to the manufacturer's protocol. Sequencing was performed in the automated Ion Chef system and loaded on the Ion PI Chip Kit v3 (Life Technologies). Data from the Proton runs were processed using Ion Torrent platform‐specific pipeline software. The threshold for a substantial gene expression change was a fold change >2.0 or <0.5 in both siTINCR1/siSCR and siTINCR2/siSCR. The fold change was calculated as the average siTINCR1 or siTINCR2 divided by the average of the siSCR.

### 2.10 RNA in situ hybridization (RNA‐ISH)

RNA in situ hybridization was performed using RNAscope (Advanced Cell Diagnostics (ACD)). Human lungs were fixed with 10% neutral‐buffered formalin for 18–32 h. Lungs were paraffin‐embedded, and 5 um tissue sections were mounted on slides. Slides were baked for 1 h at 60°C, deparaffinized in xylene and dehydrated in 100% ethanol. Sections were treated with hydrogen peroxide (ACD 322381) for 10 min at room temperature, then heated to mild boil (98–102°C) in 1x target retrieval reagent buffer (ACD 322001) for 15 min. Protease Plus (ACD 322381) was applied to sections for 30 min at 40°C in HybEZ Oven (ACD). Hybridization with target probes (HsTINCR; ACD 437891), preamplifier, amplifier, labels, and wash buffer (ACD 320058) was done according to ACD instructions. Parallel sections were incubated with ACD positive (HsPPIB; ACD 313901, MnPpib; ACD 313911) and negative (DapB; ACD 310043) control probes. For co‐staining with immunohistochemistry (IHC), IHC was performed with the detection system (ImmPRESS™: Vector laboratories). Samples were incubated with FOXJ1 (cat. No. 14‐9965‐80. Thermo Scientific, MA, USA) antibodies 30 min at room temperature. The immune reaction was detected incubating for 30 min at room temperature peroxidase‐labeled secondary reagent, ImmPRESS™ (MP‐7402 for mouse antibodies).

For fluorescent ISH (FISH), we applied PLISH (PLISH: proximity ligation‐ in situ hybridization) protocol (Nagendran et al., 2018).

### 2.11 RNA immunoprecipitation (RIP)

RIP was performed using the RiboCluster profliler^TM^ RIP‐Assay Kit (MBL, Japan) according to the manufacturer's protocols. Total RNAs and those precipitated with IgG (isotype control) and anti‐STAU1 (MBL, Japan; RN012P) antibodies (15 µg) were incubated with the supernatant. Precipitated RNAs were separated using the protein G agarose and then quantified using qRT‐PCR.

### 2.12 Statistical analysis

We used the Mann–Whitney *U* test or an unpaired t test for comparisons between two groups. Data are presented as means ± SEM or box and whiskers plot. The graphs were produced using GraphPad Prism 7.0 software. *p* values <0.05 were considered statistically significant.

## 3 RESULTS

### 3.1 TINCR localizes mainly to FOXJ1+ bronchial epithelial cells in the lung

**FIGURE 1:**
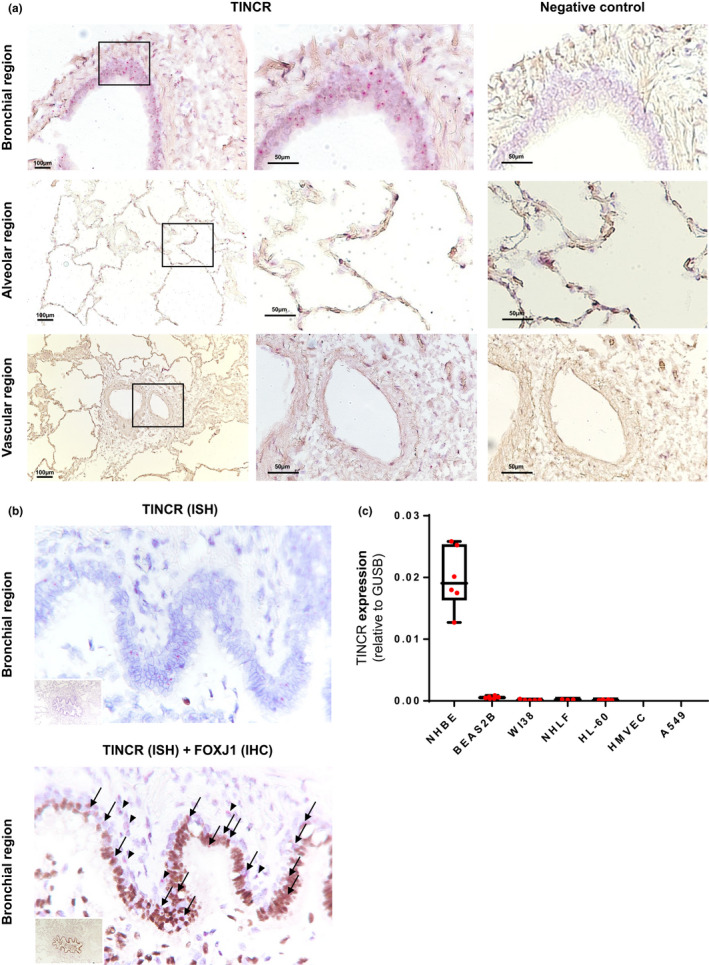
TINCR in mainly expressed in bronchial epithelial cell in human lung. (a) RNA‐ISH analysis of representative human lung tissue samples from normal histology lung. TINCR expression in normal histology lung (right image; ×100, left image; ×200–400). Boxed regions are shown enlarged on the middle and as insets on the left. (b) RNA‐ISH and co‐staining with IHCs for FOXJ1 in human lung samples. Black arrows point to double positive cells for TINCR and FOXJ1; black arrowheads indicate TINCR positive cells (FOXJ1 negative). (c) qRT‐PCR for RNA extracted from various human lung cells; NHBEC (normal human bronchial epithelial cell), BEAS2B (immortalized normal bronchial epithelial cell line), NHLF (normal human lung fibroblasts), WI38 (normal human embryonic lung fibroblast), HL‐60 (promyelocytic human cell line), HMVEC (human lung microvascular endothelial cell) and A549 (adenocarcinomic human alveolar basal epithelial cell). Data are presented as box–whisker plots, *n* = 6

To study the distribution of TINCR in the human lung, RNA‐ISH was conducted in normal histology controls. RNA‐ISH revealed that TINCR is mainly expressed in bronchial epithelial cells (Figure 1a). There was no or little expression of TINCR in alveolar, stromal, and vascular regions. Co‐staining of RNA‐ISH (TINCR; red) with IHC (FOXJ1; brown) showed that most of TINCR positive cells were FOXJ1 positive (76.2 ± 5.6%), suggesting that TINCR is mainly localized in ciliated cells (Figure 1b). We quantified the expression of TINCR in various human cell lines by qRT‐PCR, and TINCR was only detected in primary NHBECs (Figure1c).

### 3.2 TINCR is associated with cell adhesion, cell migration, extracellular matrix production, wound healing, and cell differentiation

**FIGURE 2:**
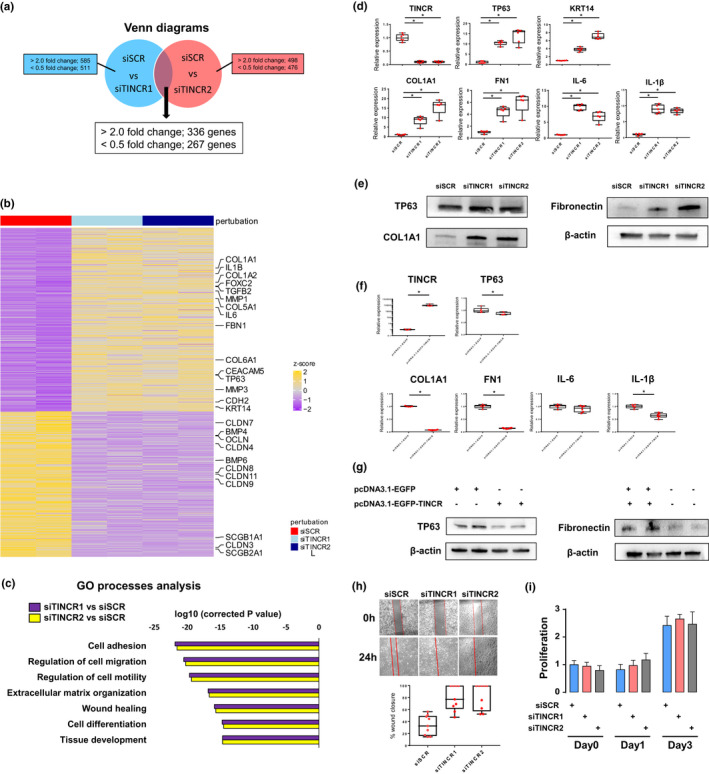
TINCR is involved in cell adhesion, cell migration, extrasellar matrix production, wound healing, tissue development and cell differentiation in human bronchial epithelial cells. (a) Venn diagram showing genes that are differentially expressed (>2.0 or <0.5 fold change) in NHBECs transfected with siTINCR1 or siTINCR2 compared to NHBECs transfected with siSCR. (b) Heat map representing color‐coded expression levels of differentially expressed 603 genes in NHBECs transfected with siTINCR1 or siTINCR2 compared to NHBECs transfected with siSCR. Increased genes are presented on the heat map in yellow and decreased genes in purple (c) Bar plots of the *p* value for GO term enrichment (GO Process) for differentially expressed genes. (d) qRT‐PCR data; and (e) Western blot analysis in NHBECs transfected with siSCR, siTINCR1 or siTICNR2. *p < 0.05. NHBECs were cultured in 6 well plate for 4 days after transfection. Data presented are from one of two independent experiments with similar results. (f‐g) qRT–PCR and western blot analysis of TINCR, TP63, COL1A1, FN1, IL‐6, and IL1β expression for NHBECs transfected with pcDNA3.1‐EGFP‐Blank or pcDNA3.1‐EGFP‐TINCR. *p < 0.05. Data presented are from one of two independent experiments with similar results. (h) Representative image of scratch cell migration assay for NHBECs transfected with siSCR or siTINCR at 0 and 24 h. The red solid lines define the areas lacking cells. The rate of migration was measured by quantifying the total distance that the cells moved from the edge of the scratch toward the center of the scratch. Ten randomly selected fields were measured for the rate of migration and compared between siSCR and siTINCR. *p < 0.05. (i) Cell proliferation was measured by WST‐1 assay for NHBECs transfected with siSCR or siTINCR

To explore the role of TINCR in human bronchial epithelial cells, we silenced TINCR in NHBECs for 96 h and identified TINCR regulated‐genes by RNA‐sequencing analysis. TINCR silencing resulted in the differential expression of 603 genes (Figure 2a and b, Table S1). Gene ontology (GO) analysis of differentially expressed genes was enriched for cell adhesion (CLDN3, CLDN4, CLDN7, CLDN8, CLDN9, CLDN11) and migration (CDH2, TGFβ2, IL‐1β, IL‐6, IL‐6ST, IL‐33), extracellular matrix (ECM) organization/ wound healing (COL1A1, COL1A2, COL5A1, COL5A3, COL6A1, COL6A2, TGFβ2, FBN1, MMP1, MMP3, MMP10, MMP11, MMP13), tissue development and cell differentiation (TP63, KRT14, SCGB1A1, SCGB2A1, BMP4, BMP6, FOXC2, CEACAM5) (Figure 2c). As for cell differentiation‐related genes, basal cell markers (TP63, KRT14) were increased and club cell markers (SCGB1A1, SCGB2A1) were decreased. qRT‐PCR validated upregulation of basal cell markers (TP63, KRT14), ECM‐related genes (COL1A1, FN1), and cytokine genes (IL‐6, IL1β) following TINCR silencing, but did not validate downregulation of the club cell marker (SCGB1A1) because the expression of SCGB1A1 mRNA was very low when NHBECs were cultured submerged on plastic plates (Figure 2d and Figure S1). Upregulation of TP63, COL1A1, and FN1 after TINCR silencing was validated on the protein level by Western blots (Figure 2e). Gain‐of‐function analysis revealed that TINCR overexpression suppressed TP63 and FN1 expression in both mRNA and protein levels (Figure 2f and g).

RNA sequencing also revealed that loss of TINCR‐induced expression of mesenchymal markers (FN1, CDH2) and pro‐inflammatory cytokines genes (TGFβ2, IL‐1β, IL‐6, IL‐33), and decreased epithelial markers (TJP1, EPCAM, OCLN) (Figure 2b). Globally TINCR inhibition induced genes associated with migration, adhesion and differentiation (Figure 2c). Scratch assay revealed that silencing of TINCR‐induced cell migration but did not alter cell proliferation (Figure 2h and i). Because loss of TINCR‐induced extracellular matrix organization, migration, and wound healing related genes, we hypothesized that TINCR may be involved in the response to lung injury and regeneration. RNA‐sequencing data from normal lung, COPD, and fibrotic lung showed that TINCR is downregulated in both COPD and fibrotic lung compared to normal lung (Figure S2a). Fibrotic lungs also showed higher expression of TP63, SCGb1A1, FOXJ1, MUC5B, SOX2, and JAG1. In addition, the TINCR homolog in mice was decreased in bleomycin‐induced fibrotic lung model (Figure S2b). These data support that downregulation of TINCR may be associated with lung injury and regeneration. Because aberrant bronchiolization is frequently observed in end‐stage IPF lung, loss of TINCR may contribute or reflect the excessive differentiation and aberrant bronchiolization.

### 3.3 Loss of TINCR expression induces aberrant bronchial cell differentiation

**FIGURE 3:**
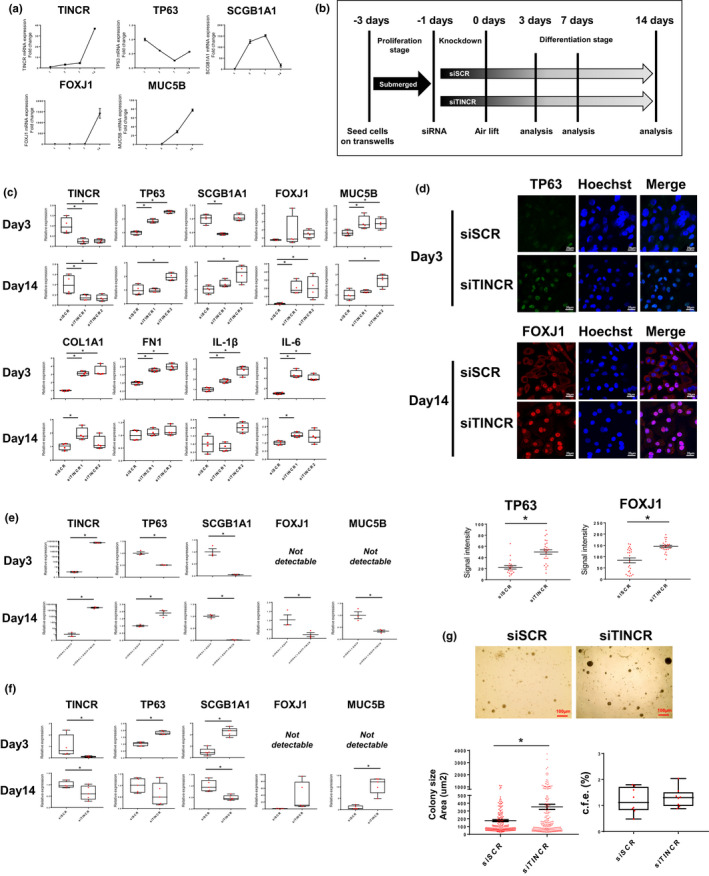
Loss of TINCR expression induced aberrant bronchial cell differentiation in ALI culture model and 3D organoid formation assay. (a) qRT–PCR analysis of relative TINCR, TP63, SCGB1A1, FOXJ1 and MUC5B expression (mean ± SEM) (b) Schematic overview of NHBECs differentiation with transfection of siRNA in ALI culture model. NHBECs were seeded in transwell inserts and expanded for 2–3 days until confluency was reached. NHBECs were transfected with siSCR or siTINCR at day‐1, subsequently airlifted and basolateral media was exchanged to differentiation medium at day0. The medium was renewed every 2 days and samples were taken for analysis at 3, 7, and 14 days after airlift. (c) qRT–PCR analysis of TINCR, TP63, SCGB1A1, FOXJ1, MUC5B, COL1A1, FN1, IL‐1β and IL‐6 expression at day3 and day14 after airlift with siRNA. *p < 0.05. Data presented are from one of two independent experiments with similar results. (d) Immuofluorescence staining of TP63 and FOXJ1 for NHBEC transfected with siSCR and siTINCR day day3 and day14. Green represents TP63, and red represents FOXJ1 staining. Blue represents nuclear DNA staining by Hoechst33342; bars =20 μm. Quantitative analysis of the singal intensity per cell per group. Bars represent mean score ± SEM.; *p < 0.05. (e) 3D organoid formation assay for NHBECs transfected siSCR or siTINCR (day14). qRT–PCR analysis of relative gene expression of TINCR, TP63, SCGB1A1, FOXJ1 and MUC5B at day14. *p < 0.05. Data presented are from one of two independent experiments with similar results. (f) Representative image of bronchial cell organoid in NHBECs transfected siSCR or siTINCR; bars =100 μm. (g) Scatter dot plot of organoid size (left) and box plot of colony formation efficacy (right). *p < 0.001. Data presented are from one of two independent experiments with similar results. (h) qRT–PCR analysis of relative TINCR, TP63, SCGB1A1, FOXJ1 and MUC5B expression at day3 and day14 after airlift with transfection of the plasmids. *p < 0.05. Data presented are from one of two independent experiments with similar results

To determine whether TINCR is essential for bronchial epithelial cell differentiation, we employed ALI culture model and 3D organoid formation assay. In ALI culture model, TP63 and SCGB1A1 were increased in the early phase (Day 1–3), and FOXJ1 and MUC5B were increased in the late phase (Day 7–14) (Figure3a). The TINCR expression was highly induced in the late phase of differentiation. The gene expression changes during differentiation of NHBECs in the ALI culture model were validated in a dataset from different sources (Figure S3a) (Ross et al., 2007). Next, we performed a loss‐of‐function experiment in NHBECs with the ALI culture model. NHBECs were transfected with siRNA and subjected to air‐lift on the next day, and gene expression was evaluated by qRT‐PCR in the early phase (day3) and late phase (day 7–14) (Figure 3b). In siTINCR groups, basal cell TP63 and KRT14 were significantly induced during the early phase, but FOXJ1 was significantly induced during the late phase (Figure 3c and d, Figure S3b). Interestingly, the loss of TINCR also induced extracellular matrix‐related genes (COL1A1, FN1) and proinflammatory cytokine genes (IL‐1β, IL‐6) during the early phase of ALI culture model, but not in the late phase. We also evaluated the impact of TINCR overexpression on bronchial cell differentiation in the ALI model. TINCR overexpression suppressed basal cell phenotype (TP63) and club cell differentiation in the early phase and ciliated and goblet cell differentiation in the late phase (Figure 3e).

To validate these results, we also employed 3D organoid formation assay. We silenced TINCR in NHBECs and plated these cells on the matrigel, and gene expression was evaluated by qRT‐PCR in the early and late phase. Loss of TINCR‐induced TP63 in the early phase and FOXJ1 in during the late phase, which was consistent with the results in the ALI culture model (Figure 3f, Figure S3c). SCGB1A1 was higher in the early phase and lower in late phase, whereas MUC5B higher in the late phase. Morphologically, loss of TINCR‐induced larger organoid size, which is consistent with the more differentiated state of bronchial cells (Figure 3h). Organoid formation efficacy was not affected by TINCR loss (Figure 3h). Taken together, TINCR regulates basal cell markers in the early phase and ciliated cell marker in the late phase during differentiation in both the ALI culture model and 3D organoid formation assay. This suggests that the role of TINCR is to prevent aberrant and excessive bronchial cell differentiation, and bronchial cells stop differentiation toward ciliated cells by increasing TINCR expression.

### 3.4 TINCR regulates critical transcription factors of bronchial cell differentiation

**FIGURE 4:**
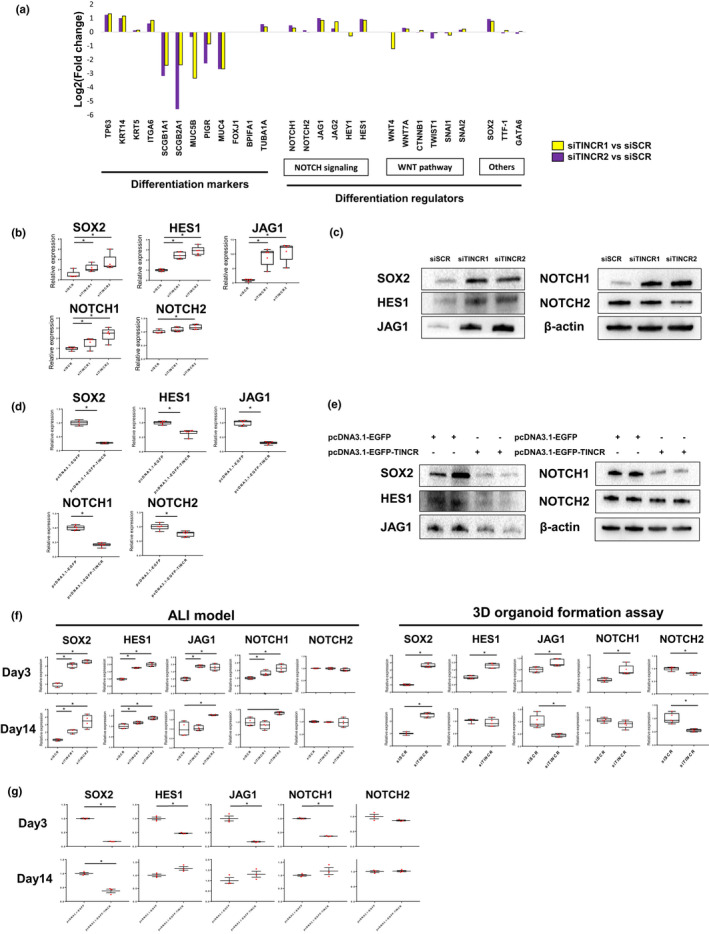
(a) Selected gene expressions regarding epithelial mesenchymal markers, differentiation makers and regulators from RNA‐sequencing data. (b and c) qRT–PCR and western blot analysis of SOX2, HES1, JAG1, NOTCH1 and NOTCH2 expression at day4 after siRNA. *p < 0.05. Data presented are from one of two independent experiments with similar results. (d and e) qRT–PCR and western blot analysis of SOX2, HES1, JAG1, NOTCH1 and NOTCH2 expression for NHBECs transfected with pcDNA3.1‐EGFP‐Blank or pcDNA3.1‐EGFP‐TINCR. *p < 0.05. Data presented are from one of two independent experiments with similar results. (f) qRT–PCR analysis of SOX2, HES1, JAG1, NOTCH1 and NOTCH2 expression at day3 and day14 after airlift with siRNA and 3D organoid matrigel. *p < 0.05. Data presented are from one of two independent experiments with similar results. (g) qRT–PCR analysis of SOX2, HES1, JAG1, NOTCH1 and NOTCH2 expression at day3 and day14 after airlift with transfection of pcDNA3.1‐EGFP‐Blank or pcDNA3.1‐EGFP‐TINCR

To examine how TINCR alter differentiation state of bronchial epithelial cell, we focused on critical regulators of differentiation. Previous studies have shown that NOTCH signaling (ligands; JAG1, JAG2, receptors; NOTCH1, NOTCH2, downstream targets; HES1, HEY1), WNT pathway (WNT4, WNT7A, CTNNB1, SNAI1, SNAI2) and transcription factors (SOX2, TTF‐1, GATA6) influences genetic programs for bronchial cell differentiation (Costa & Lim, 2001; Gomi et al., 2015; Gontan et al., 2008; Schmid et al., 2017; Tsao et al., 2016). We therefore hypothesized that TINCR may regulate these critical regulators of differentiation. In our sequencing data, SOX2, JAG1, HES1, and NOTCH1 were not included in the differentially expressed gene list, however, they are markedly induced in siTINCR‐treated NHBECs (Figure 4a), which were validated by qRT‐PCR and western blot assay (Figure 4b and c). Conversely, overexpression of TINCR suppressed SOX2, JAG1, HES1, and NOTCH1 in both mRNA and protein expression levels (Figure 4d and e). Moreover, these genes were highly induced in the early phase of bronchial cell differentiation by loss of TINCR in both the ALI culture model and 3D organoid formation assay (Figure 4f). The upregulations of SOX2 continued until day14 in both models (Figure S3b). Moreover, overexpression of TINCR suppressed SOX2 expression throughout differentiation (day 4–14) (Figure 4g). Therefore, we concluded that TINCR regulates SOX2 and NOTCH signaling genes (NOTCH1, HES1, and JAG1) during the bronchial cell differentiation.

### 3.5 TINCR induces mRNA decay to maintain the normal differentiation state of bronchial epithelial cells partially in corporation with STAU1

**FIGURE 5:**
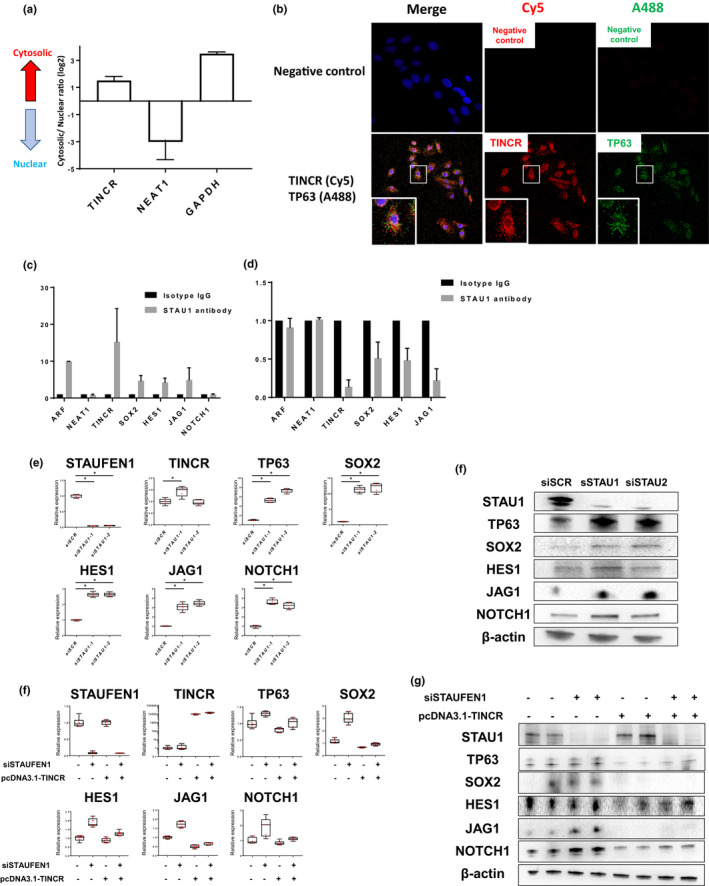
TINCR binds to STAU1 protein and controls critical regulators of differentiation. (a) Bar graphs show percentage of TINCR in the cytoplasm (black) and nucleus (white). NEAT1 serves as a positive control for nucleus enriched RNA and GAPDH serves as a positive control for cytoplasmic RNA. Data presented are from two independent experiments. (b) RIP experiments were performed using isotype IgG and STAU1 antibody to immunoprecipitated STAU1 protein/mRNAs complexes in total‐cell extracts of NHBECs, and relative enrichment was determined as RNA associated with STAU1 IP relative to an input control. Relative ARF1 enrichment served as a positive control and NEAT1 as a negative control as NEAT1 does not interact with STAU1. Data presented are from two independent experiments. (c) Relative mRNA enrichment of STAU1 antibody in total‐cell extracts of NHBECs transfected with siSCR or siTICNR. Data presented are from two independent experiments. (d and e) qRT–PCR and western blot analysis of TINCR, TP63, SOX2, HES1, JAG1, NOTCH1 and NOTCH2 expression for NHBECs transfected with siSCR or siSTAU1. NHBECs were seeded on 6 well plate at 2 × 10^5^ density and analyzed at day4 after transfection of siRNA reagents. *p < 0.05. (f and g) qRT–PCR and western blot analysis of TINCR, TP63, SOX2, HES1, JAG1, NOTCH1 and NOTCH2 expression for NHBECs transfected with siSCR or siSTAU after transfection of pcDNA3.1‐EGFP‐Blank or pcDNA3.1‐EGFP‐TINCR for 4 h. NHBECs were seeded on 24 well plate at 1 × 10^5^ density and analyzed at day4 after transfection of siRNA reagents

Previous studies have shown that TINCR is a predominantly cytoplasmic lncRNA and together with STAU1 protein degrades target mRNAs, which is called as STAU1‐mediated mRNA decay (Park, 2013; Xu et al., 2015, 2017). We hypothesized that TINCR binds to STAU1 and controls the mRNA expression of critical differentiation regulators including SOX2 and NOTCH signaling genes through STAU1‐mediated mRNA decay in bronchial epithelial cells. Therefore, we investigated the subcellular localization of TINCR and the association between TINCR, STAU1, and mRNAs of differentiation regulators. To detect the distribution of TINCR in bronchial epithelial cells, we fractionated NHBECs into nuclear and cytoplasmic fractions and analyzed RNA abundance by qRT‐PCR. TINCR was mainly expressed within the cytoplasm (86%) in NHBECs (Figure 5a), consistent with previous reports (Kretz et al., 2013; Xu et al., 2015). FISH validated the cytoplasmic localization of TINCR (Figure 5b). We performed RNA immunoprecipitation (RIP) assay to analyze the association of STAU1 with TINCR, SOX2, HES1, JAG1, and NOTCH1. RIP assay showed a remarkable enrichment of TINCR, SOX2, HES1, and JAG1 after pull‐down with a STAU1 antibody compared to IgG control (Figure 5c). Moreover, loss of TINCR reduced the bindings between STAU1 protein and target SOX2, HES1, and JAG1 (Figure 5d). These data indicate that TINCR regulates the binding STAU1 to SOX2, HES1, and JAG1. To determine whether STAU1 affects these transcription factors, we conducted a loss of function analysis for STAU1 in NHBECs. Loss of STAU1 induced the expression of TP63, SOX2, HES1, JAG1, and NOTCH1, however, it did not affect TINCR expression (Figure 5e). The protein expression of TP63, SOX2, HES1, JAG1, and NOTCH1 was also induced by loss of STAU1, indicating that STAU1 protein plays an important role in controlling differentiation regulators (Figure 5f).

We performed a co‐transfection of siSTAU1 and pcDNA3.1‐TINCR plasmid to study the reversal effect of TINCR overexpression. TINCR overexpression did not completely rescued the increased expression of TP63, SOX2, HES1, JAG1, and NOTCH1 induced by STAU1 silencing (Figure 5f and g). These data suggest that TINCR partially suppresses the expressions of SOX2, HES1, and JAG1 mRNAs through STAU1‐mediated mRNA decay. However, the decay of these mRNAs is mainly dependent on TINCR expression. Therefore, we hypothesized that TINCR itself binds to target mRNAs directly and affects their expressions in corporation with STAU1 (Kretz et al., 2013). To evaluate the direct binding of TINCR to target mRNAs, we investigated the complementary base pairing with TINCR motifs in SOX2, HES1, JAG1, and NOTCH1 mRNAs, using BLAST (https://blast.ncbi.nlm.nih.gov/). Although SOX2, JAG1, and HES1 did not have complimentary base pairing with TINCR motif, NOTCH1 partially formed complementary base pairing with TINCR motifs, suggesting that TINCR together with STAU1 induces NOTCH1 mRNA decay (Figure S4). We could not clarify the exact mechanism how TINCR controls the expression of SOX2, JAG1, and HES1. The regulation of these genes by TINCR may be controlled by several different mechanisms.

## 4 DISCUSSION

**FIGURE 6:**
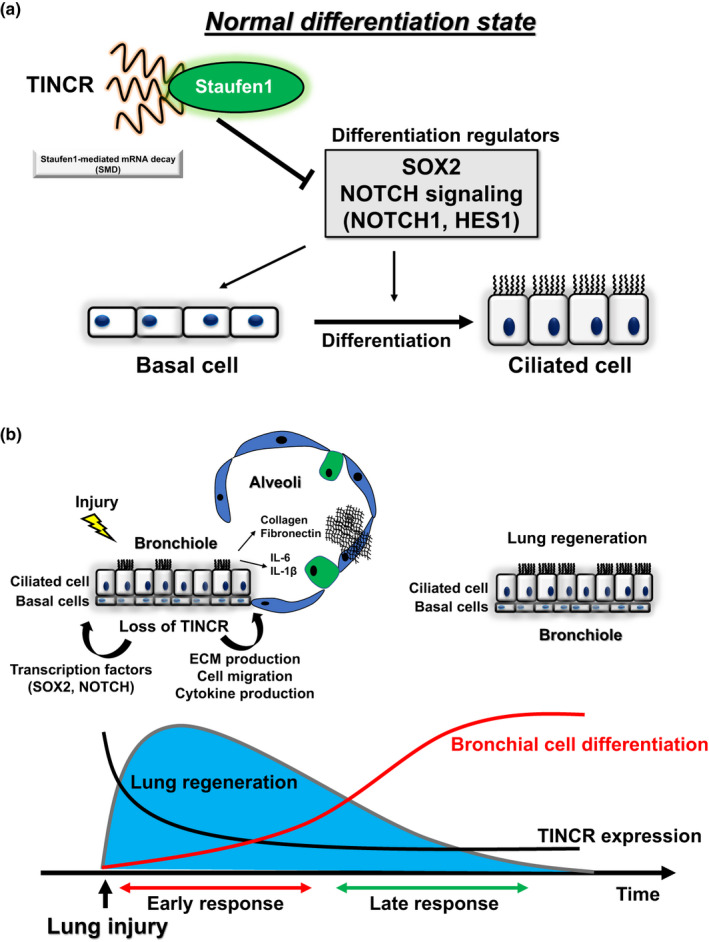
(a) A schematic diagram of the regulatory role of lncRNA TINCR in bronchial cell differentiation. TINCR together with STAU1 controls the expression of critical regulators of bronchial cell differentiation (SOX2 and NOTCH signaling genes) and maintains the normal differentiation state. (b) Proposed model representing that loss of TINCR induces aberrant bronchial differentiation and lung regeneration. In early response to lung injury, loss of TINCR promote lung regeneration and in late response aberrant differentiation of bronchial cells

In this study, we identified novel roles for TINCR, an epithelial lncRNA in maintaining the differentiation state of bronchial epithelial cells. In the mammalian lung TINCR is restricted to mainly FOXJ1 expressing bronchial epithelial cells, decreased after injury and present mainly in the cytoplasm. Inhibition of TINCR leads to an early basal cell cellular phenotype followed by ciliary cell differentiation (Figure 6a). We also demonstrated that TINCR affects the expression of critical regulators of differentiation such as SOX2, JAG1, and HES1 partially through binding to STAU1 protein. These findings provide new insights into the molecular mechanisms coordinating bronchial cell differentiation programs.

One of the major findings of this study was that TINCR controls the differentiation state of bronchial epithelial cells. Especially, TINCR terminates the differentiation toward ciliated cells and may prevent aberrant and excessive differentiation. Multiple regulatory factors such as small noncoding RNAs and transcription factors coordinate bronchial cell differentiation (Costa & Lim, 2001; Perdomo et al., 2013). Our results showed that TINCR controls both NOTCH signaling and SOX2 expression during differentiation. NOTCH signaling and SOX2 are the critical regulators of differentiation. In particular, SOX2 is important for both maintaining basal cell phenotype and inducing ciliated cell differentiation, which is consistent with our results (Tompkins et al., 2009). SOX2 directly regulates the expression of TP63, resulting in the differentiation of bronchial epithelial cells into basal cells (Ochieng et al., 2014). NOTCH signaling also regulates differentiation toward ciliated cells (Gomi et al., 2015; Rock et al., 2011). Especially, NOTCH1 and NOTCH3 receptors have been reported to play critical roles in human bronchial cell differentiation (Gomi et al., 2015). These data support that TINCR regulates bronchial cell differentiation through SOX2 and NOTCH signaling. Although maintaining basal cell and inducing ciliated cell are opposing processes, the impact of TINCR loss on differentiation state may be separated into two different phases: an early basal‐like cell phenotype and a ciliated cell differentiation in the late phase.

We also found that TINCR controls critical regulators of differentiation partially through STAU1‐mediated mRNA decay. STAU1 is a cytoplasmic protein and exerts multiple functions as a posttranscriptional regulator (Park, 2013). STAU1 participates in controlling the differentiation of adipocytes, myoblasts, and epidermis (Cho et al., 2012; Kim et al., 2007; Park & Maquat, 2013). A previous study showed that STAU1 together with TINCR increased target mRNA stability of epidermis differentiation (Kretz et al., 2013). On the other hand, STAU1 induced mRNA degradation regarding adipocytes and myoblasts differentiation (Kretz, 2013). As for bronchial differentiation, we found that STAU1 binds to differentiation regulators SOX2, HES1 and JAG1 mRNA, and partially induces degradation of these mRNAs together with TINCR. However, the degradations of these mRNAs are dependent on TINCR expression. One possible explanation of this result is that TINCR itself has specific binding motifs to target mRNAs and affect their expression levels. TINCR may affect the expression levels of targets genes by itself and together with STAU1 (Kretz et al., 2013). In fact, our results showed that there was no remarkable binding of STAU1 protein to NOTCH1 mRNA, however, loss of TINCR‐ and STAU1‐induced NOTCH1 mRNA expression. Because our computational analysis indicates the binding of TINCR to NOTCH1 mRNA, there may be another mechanism of regulating NOTCH1 expression by TINCR. Further studies are needed to evaluate the association between TINCR and its target mRNAs.

An intriguing finding in our study had to do with the biphasic nature of the response to loss of TINCR in both ALI and in organoid system. Immediately after inhibition of TINCR we saw increased expression of mesenchymal markers, pro‐inflammatory cytokines, and decreased epithelial markers. One of the explanations is the induction of epithelial‐mesenchymal transition (EMT) like phenotype. NHBECs have been shown to be able to undergo EMT, which was enhanced by IL‐1ß (Câmara, 2010; White et al., 2008). Previous reports showed that TGFß2‐ and IL‐6‐induced EMT phenotype with migrative phenotype in NHBECs (Fischer, 2014; Luo et al., 2013). In addition to mesenchymal markers, loss of TINCR induced the expression of IL‐1β and IL‐6 accompanied by induction of a basal cell phenotype with increased expression of TP63. Thus, the early response to loss of TINCR constituted of dedifferentiation of airway epithelial cells, to a basal cell‐like phenotype, migration with EMT‐like phenotype and production of proinflammatory cytokines replaced by an enhanced ciliated cell differentiation. The reason for this is not completely clear, however, it could be that temporary loss of TINCR may be important for lung response to injury and the basal cell phenotype is associated with that. A previous study showed that TP63 expressing basal cells contribute to lung regeneration of alveolar compartment in mice and are capable of migrating into alveolar space and can act as progenitor cells (Guo et al., 2019; Kumar et al., 2011; Rock et al., 2010). In addition, a gene signature of basal cells was enriched in bronchoalveolar lavage from fibrotic lung disease (Prasse et al., 2019). TINCR expression was decreased in human and in mice lung injury models, therefore these could suggest that decreased expression of TINCR in bronchial cells after lung injury might contribute to lung repair by enabling basal cells to migrate into alveolar space, produce extracellular matrix and differentiate into ciliated cells to regenerate lung and bronchiole (Figure 6B). Further in vivo studies are needed to clarify the role of TINCR in lung regeneration.

In summary, TINCR has multiple biological roles in human bronchial epithelial cells controlling bronchial cell differentiation through coordination of critical differentiation regulator. Our findings provide a better understanding of the regulation of human bronchial epithelial cell differentiation.

## CONFLICT OF INTEREST

NK served as a consultant to Biogen Idec, Boehringer Ingelheim, Third Rock, Pliant, Samumed, NuMedii, Theravance, LifeMax, Three Lake Partners, Optikira, Astra Zeneca over the last 3 years, reports Equity in Pliant and a grant from Veracyte and nonfinancial support from MiRagen and Astra Zeneca. NK as IP on novel biomarkers and therapeutics in IPF licensed to Biotech.

## AUTHORS’ CONTRIBUTIONS

Conception and design: NO, NK. Acquisition, analysis, and interpretation: NO, JCS, NK. Drafting/revising work: NO, NK, FA, JCS. Final approval of submitted work: NO, KS, QL, JCS, TA, FA, MC, JD, NH, YH, NK.

## Supporting information



Supplementary Material

Supplementary Material
