# Supplementary material for: Long noncoding RNA TINCR is a novel regulator of human bronchial epithelial cell differentiation state
Source: Physiol Rep. 2021 Feb 1;9(3):e14727. doi: 10.14814/phy2.14727 (PMC7851438; doi:10.14814/phy2.14727)

Supplement Figure.S1

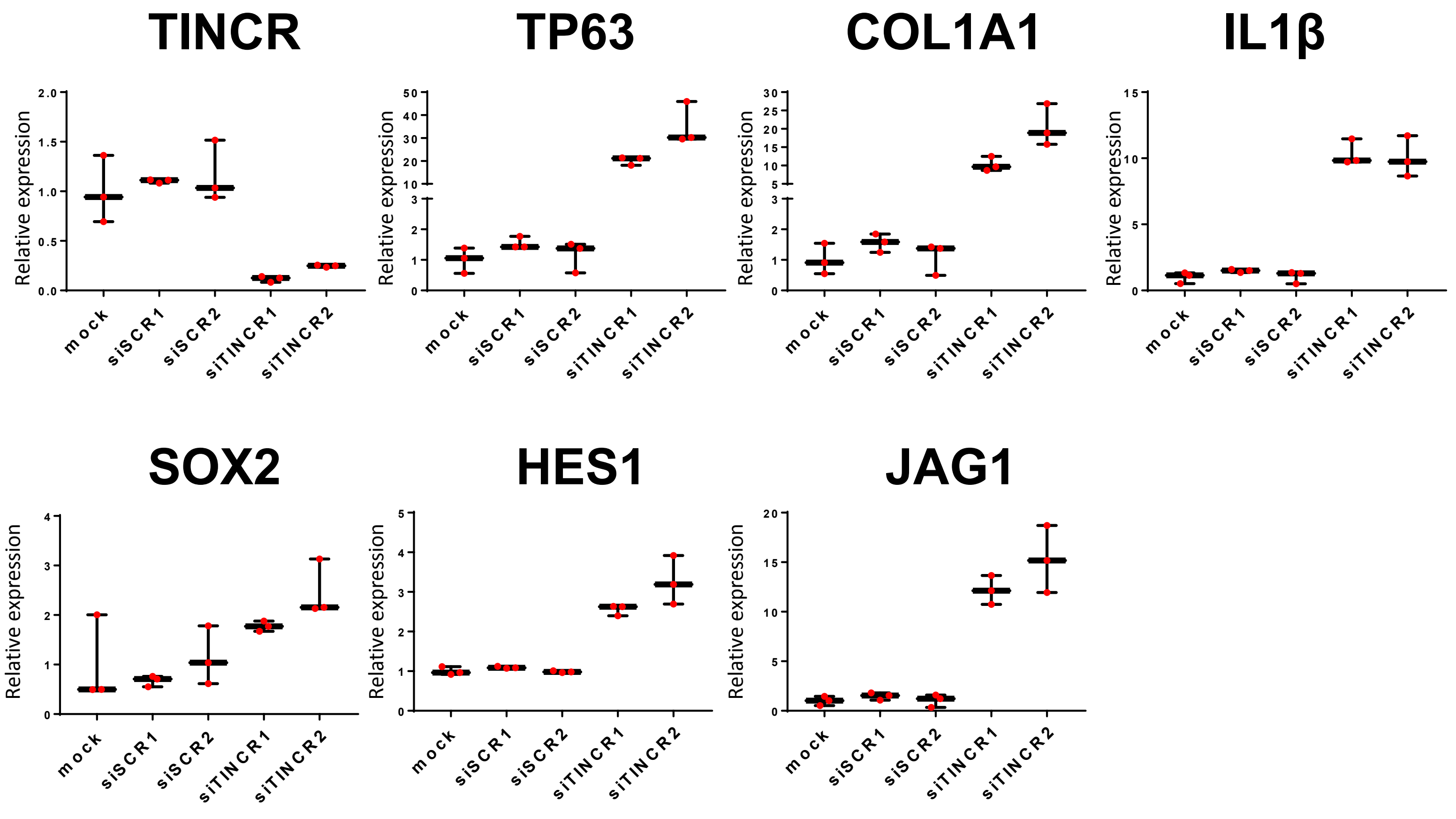

Supplement Figure.S2

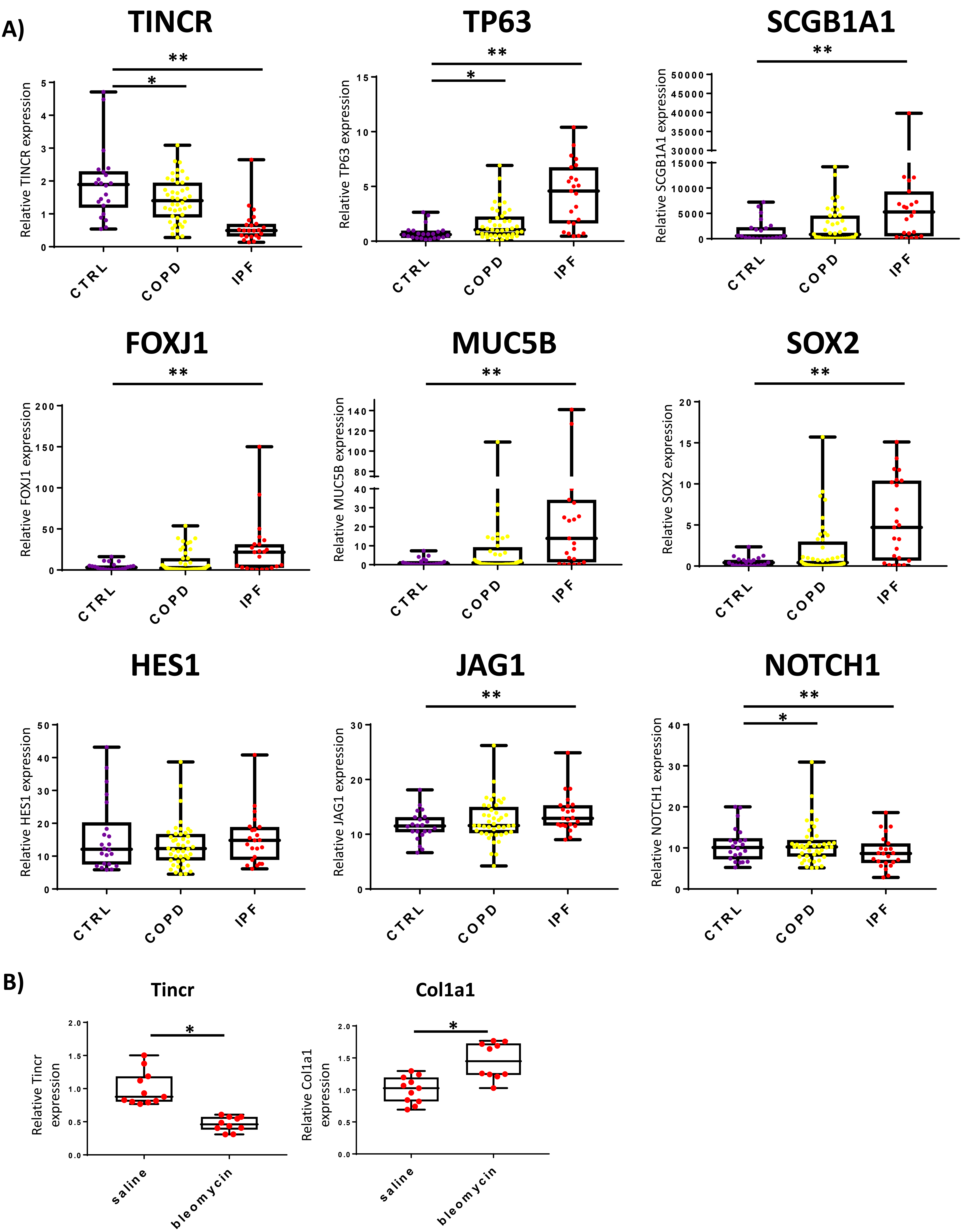

Supplement Figure.S3

A)

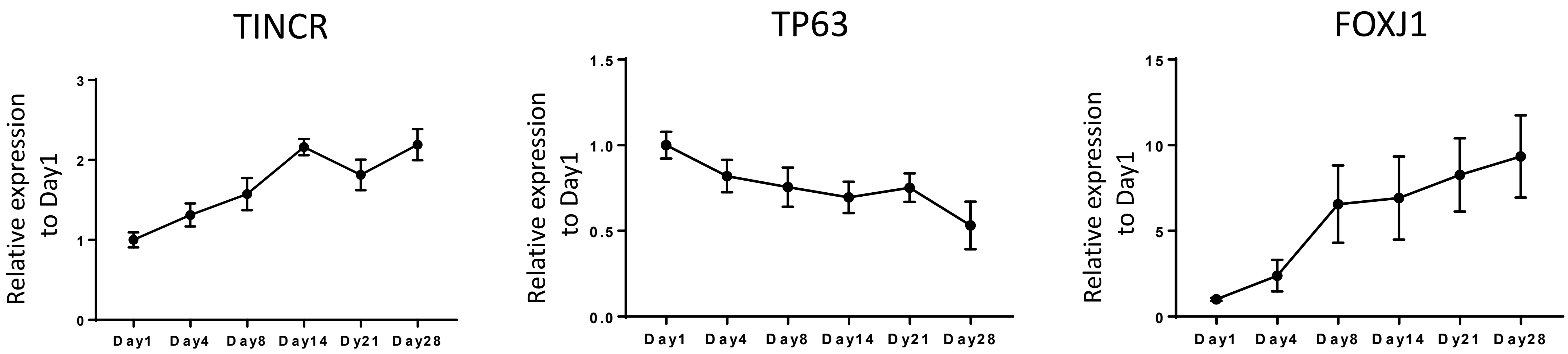

B)

ALI culture model

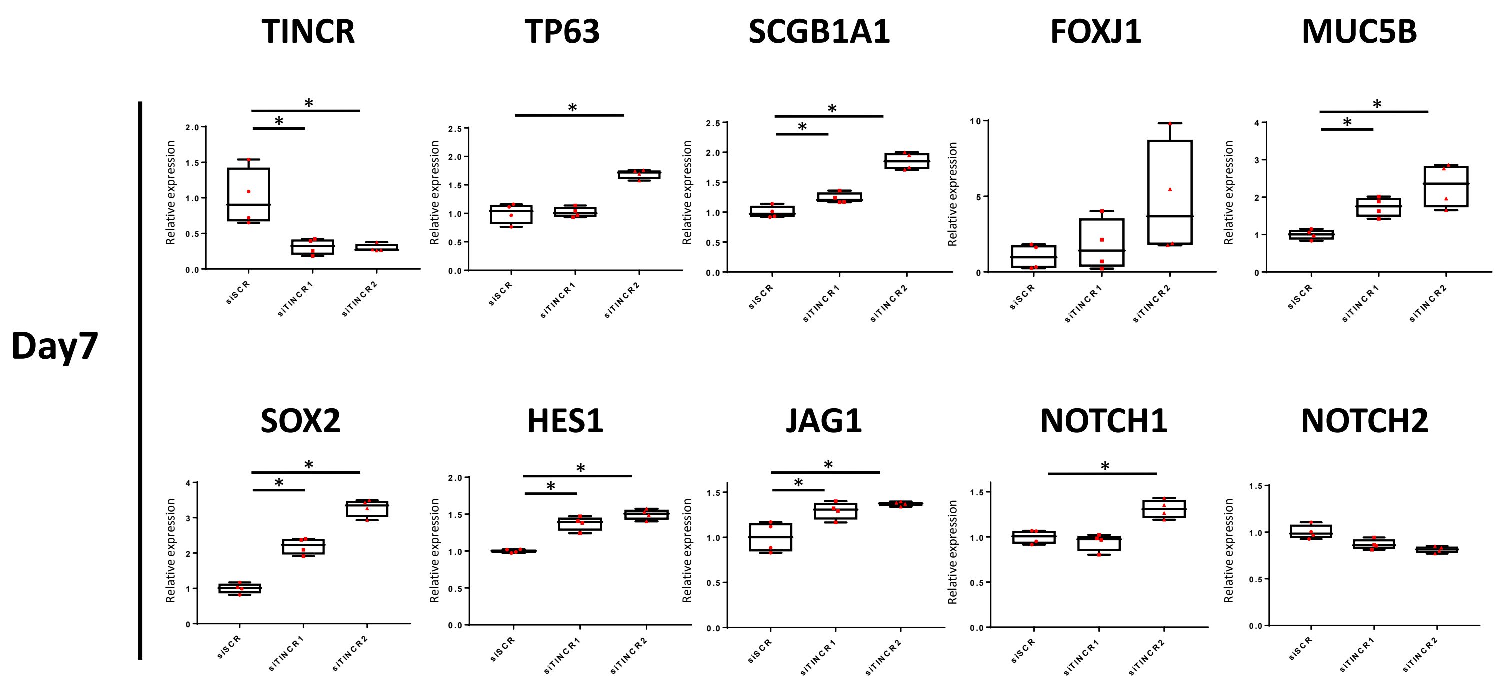

C)

3D organoid formation assay

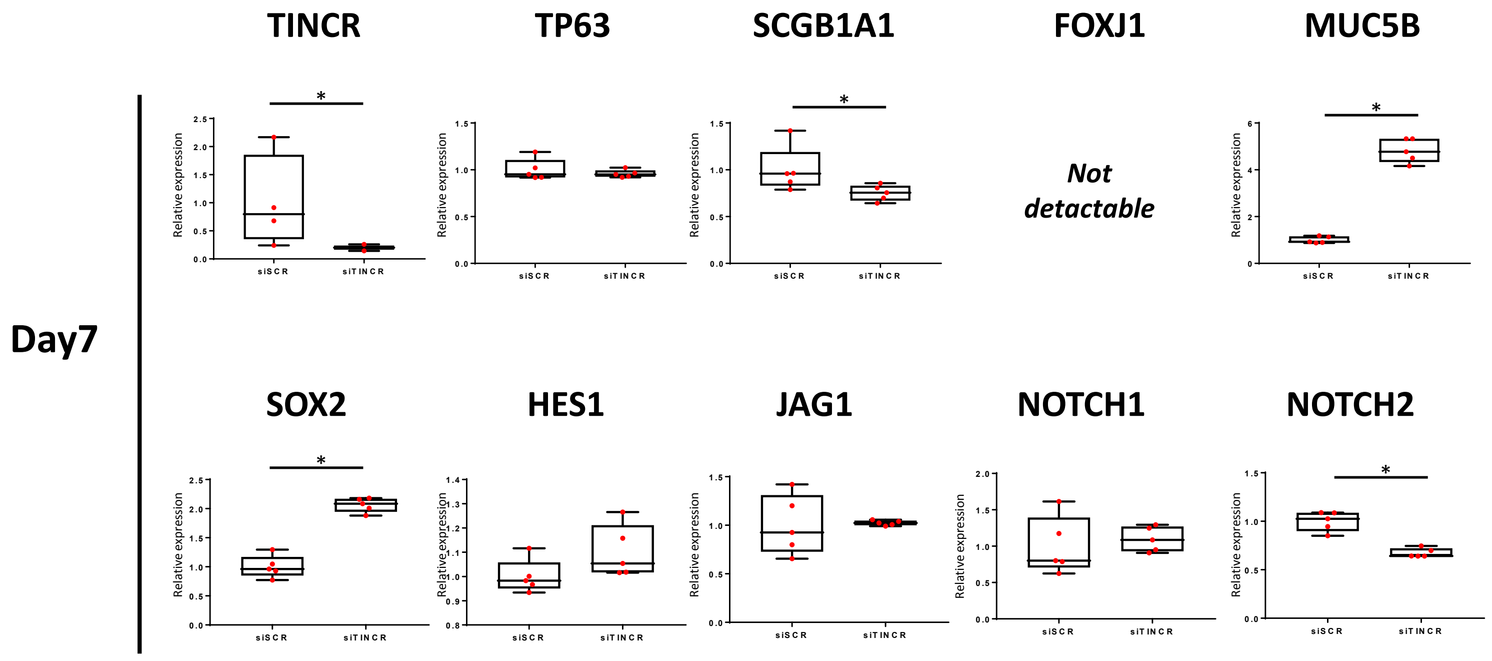

Supplement Figure.S4

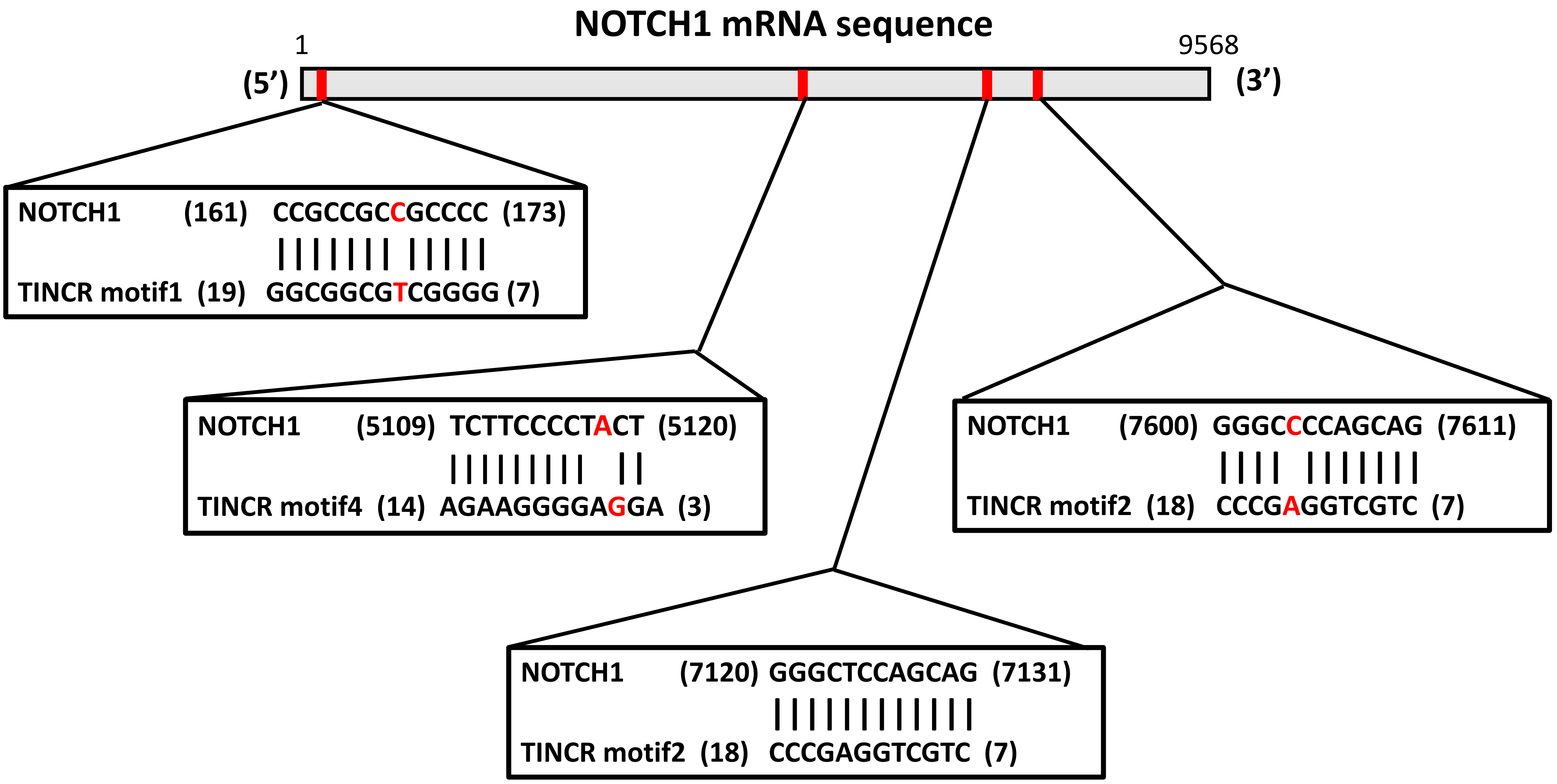

Supplement Figure.S5

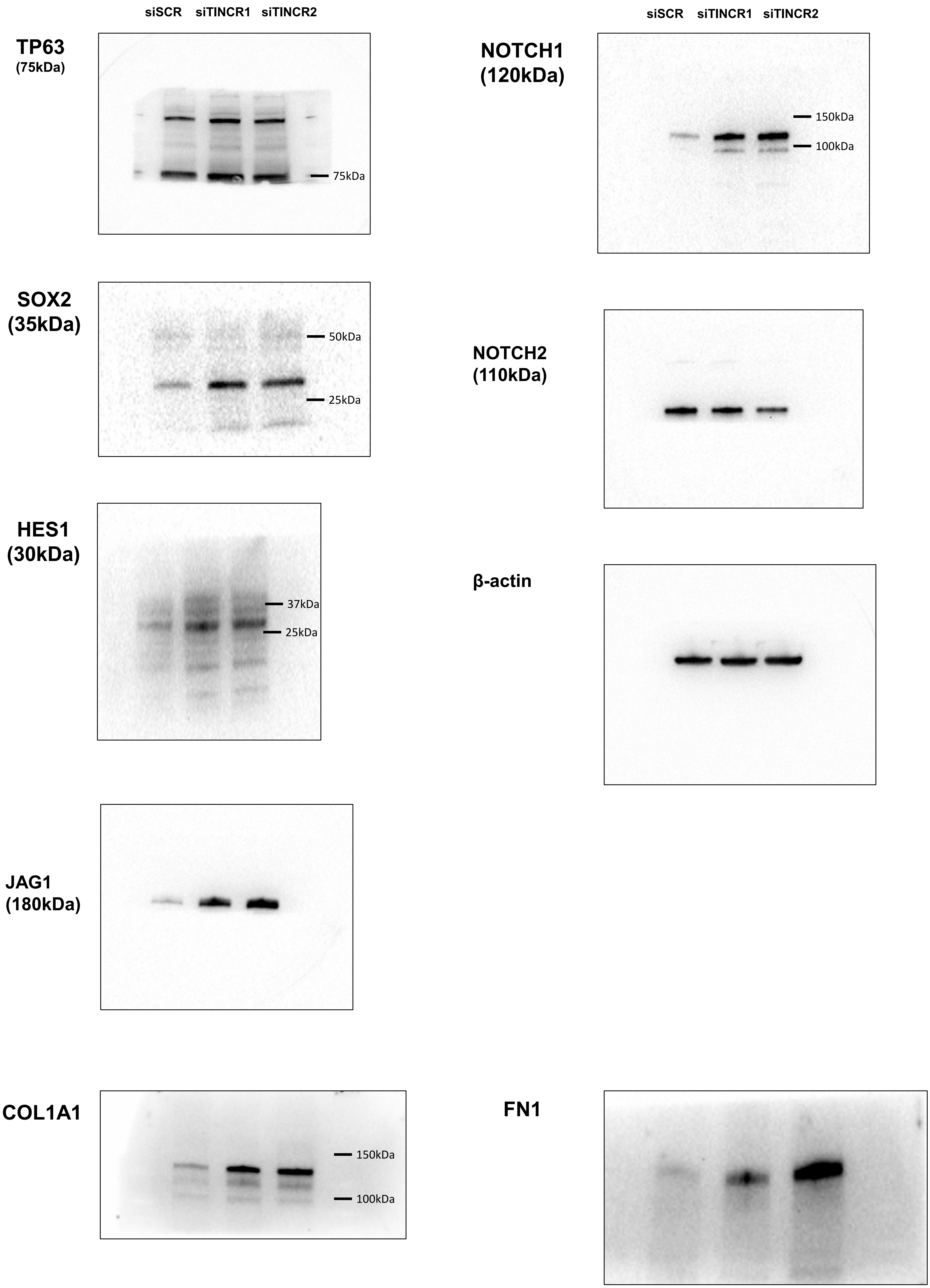

Supplement Figure.S6

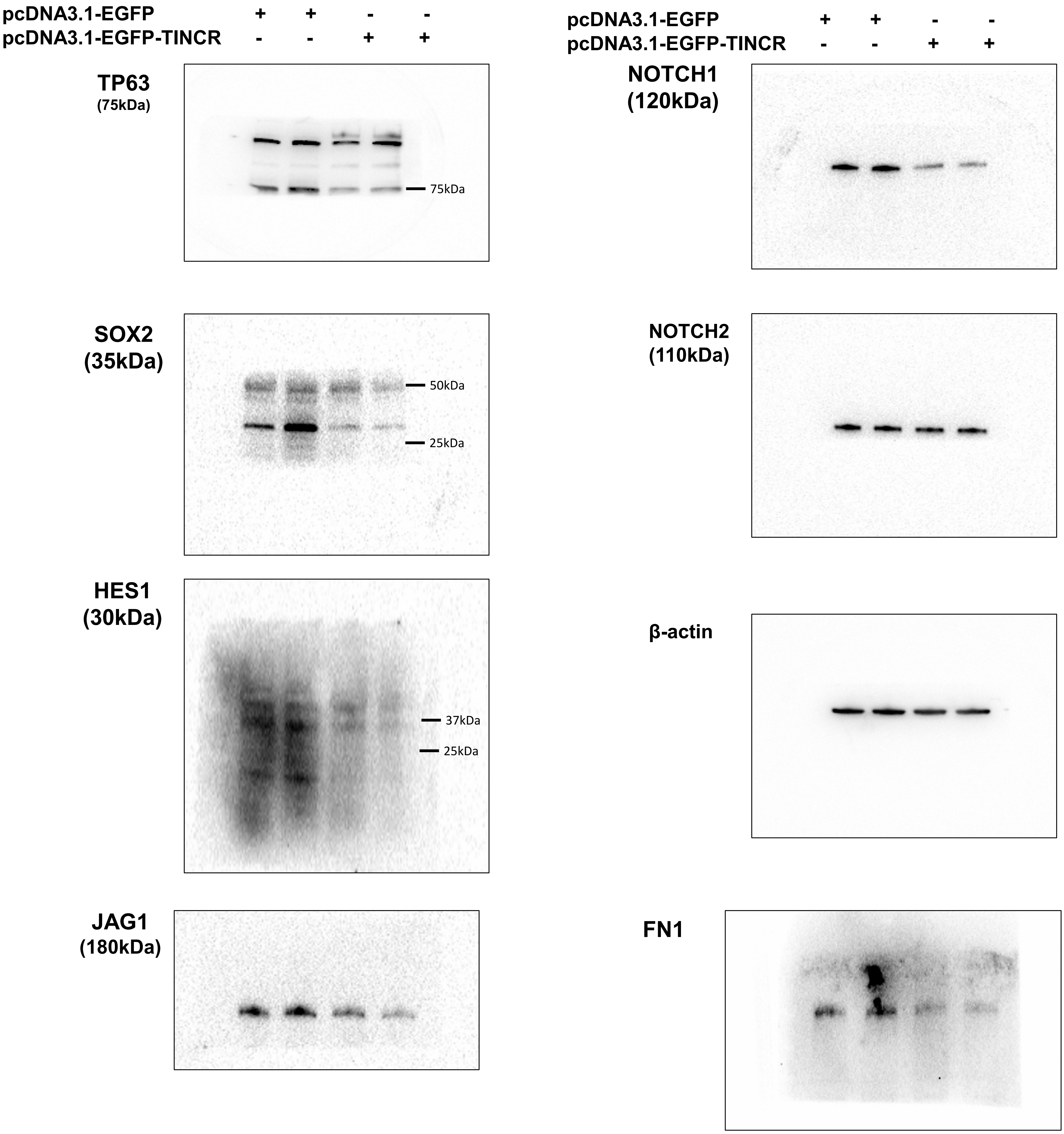

Supplement Figure.S7

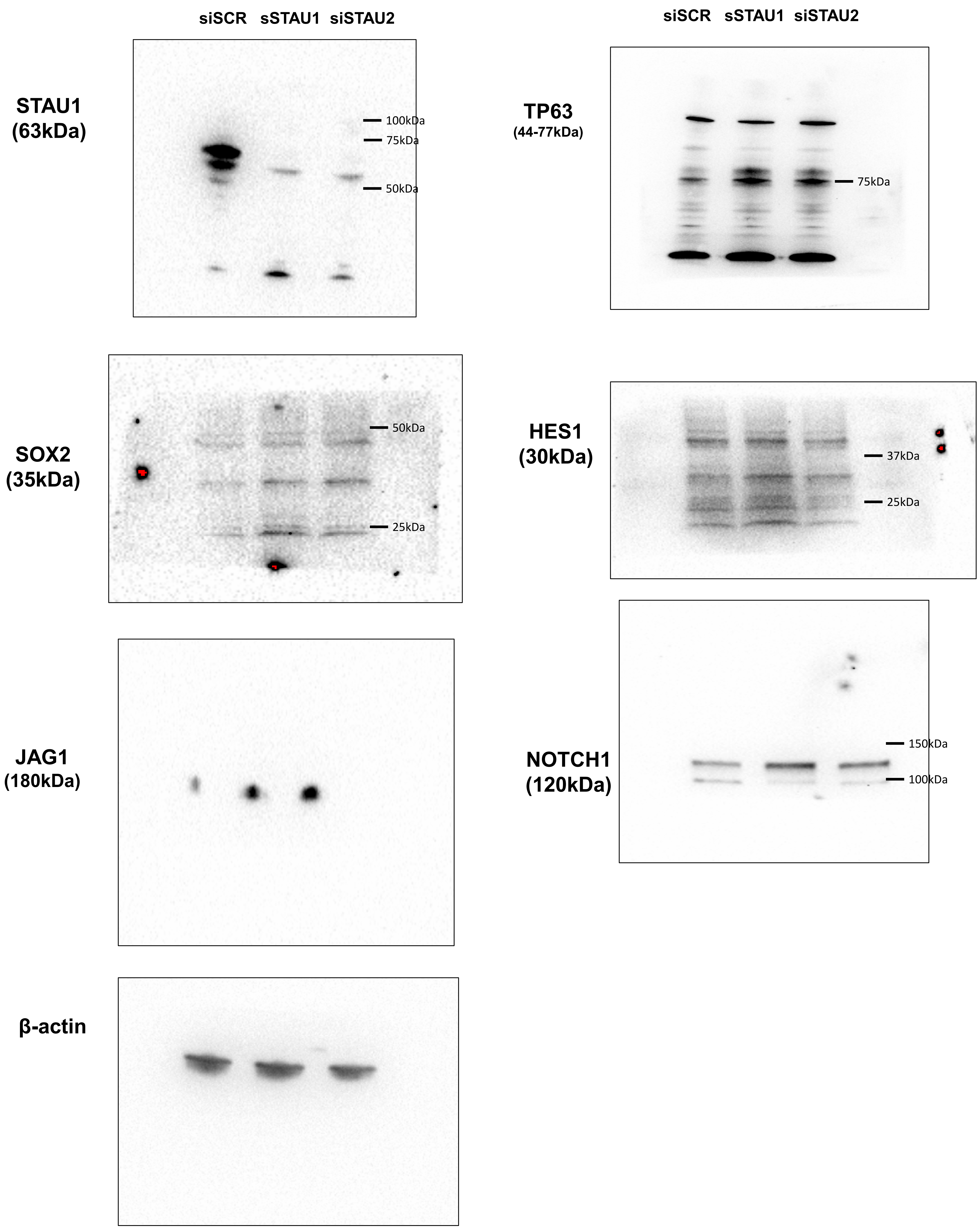

Supplement Figure.S8

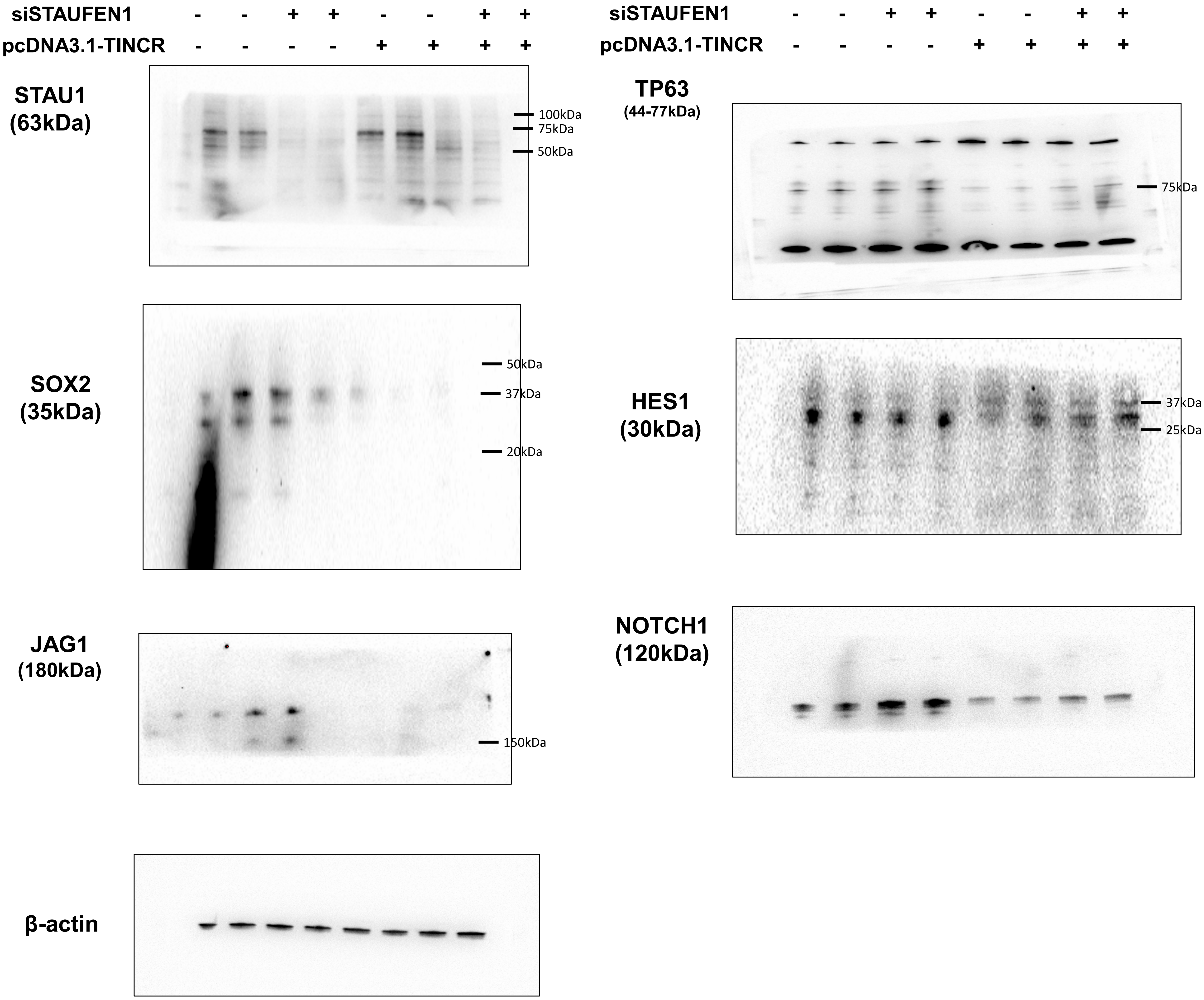

Supplement: Supplementary file 1 — Supplementary Material [file PHY2-9-e14727-s001.pdf]
