# Supplementary material for: Long noncoding RNA TINCR is a novel regulator of human bronchial epithelial cell differentiation state
Source: Physiol Rep. 2021 Feb 1;9(3):e14727. doi: 10.14814/phy2.14727 (PMC7851438; doi:10.14814/phy2.14727)
Supplement: Supplementary file 2 — Supplementary Material [file PHY2-9-e14727-s002.docx]

**Supplemental Figure S1.**

qRT-PCR data of NHBECs transfected with mock, siSCR1, siSCR2, siTINCR1 or siTICNR2. NHBECs were cultured in 6 well plate for 4 days after transfection. Data presented are from one of two independent experiments with similar results.

**Supplemental Figure S2.**

**Decreased TINCR expression in COPD and fibrotic lungs.**

(A) Box-and-whisker plots of sequencing data from IPF (n = 23), controls (CTRL; n = 22) and COPD (n = 44). * p < 0.05, ** p < 0.001. (B) The expression levels of Tincr and Col1a1 were assessed by quantitative RT-PCR in the bleomycin model of lung fibrosis (day 21) (Saline; n=11, Bleomycin; n=10). Whisper and box plot show the relative changes (fold) by setting the indicated control level to 1.0. * p < 0.01.

**Supplemental Figure S3.**

**Gene expression profiles during bronchial cell differentiation**

(A) Data from GEO dataset (GSE5264) (1). Microarray analysis was performed to identify transcriptional changes that occur during bronchial cell differentiation of NHBECs cultured at an ALI model. NHBECs from three different donors were cultured and collected at different time points from day 0 to day 28 of ALI culture.

(B) (C) qRT–PCR analysis of relative TINCR, TP63, SCGB1A1, FOXJ1, MUC5B, SOX, HES1, JAG1, NOTCH1 and NOTCH2 expression at day7 in ALI culture model (B) and 3D organoid formation assay (C). * p < 0.05.

**Supplemental Figure S4.**

**Computational analysis of complementary base pairing with TINCR motifs.**

TINCR motifs binding sites on NOTCH1 mRNA predicted by online software. TINCR motif 1, 2, and 4 have several complimentary sequences with NOTCH1 mRNA. Red characters indicate base pair mismatch.

**Supplemental Figure S5.**

Full western blot images of TP63, SOX2, HES1, JAG1, NOTCH1, NOTCH2, COL1A1, FN1 and β-actin for NHBECs transfected with siSCR, siTINCR1 or siTICNR2.

**Supplemental Figure S6.**

Full western blot images of TP63, SOX2, HES1, JAG1, NOTCH1, NOTCH2, FN1 and β-actin for NHBECs transfected with pcDNA3.1-EGFP-Blank or pcDNA3.1-EGFP-TINCR.

**Supplemental Figure S7.**

Full western blot images of TP63, SOX2, HES1, JAG1, NOTCH1, NOTCH2, and β-actin for NHBECs transfected with siSCR, siSTAU1 or siSTAU2.

**Supplemental Figure S8.**

Full western blot images of TP63, SOX2, HES1, JAG1, NOTCH1, NOTCH2, and β-actin for NHBECs transfected with siSCR or siSTAU after transfection of pcDNA3.1-EGFP-Blank or pcDNA3.1-EGFP-TINCR for 4 hours.

**References**

1. **Ross AJ DL, Brighton LE, Devlin RB.** Mucociliary differentiation of serially passaged normal human tracheobronchial epithelial cells. *Am J Respir Cell Mol Biol* Aug;37(2):169-85.: 2007.
